# Protective Effects of Dietary Vitamin D_3_, Turmeric Powder, and Their Combination against Gasoline Intoxication in Rats

**DOI:** 10.3390/ph17050619

**Published:** 2024-05-10

**Authors:** Gulfira A. Yestemirova, Zura B. Yessimsiitova, Michael Danilenko

**Affiliations:** 1 Department of Biodiversity & Bioresources, Faculty of Biology and Biotechnology, Al-Farabi Kazakh National University, Almaty 050040, Kazakhstan; yestemirova.gulfira@kaznu.kz (G.A.Y.); zura@kaznu.kz (Z.B.Y.); 2 Department of Clinical Biochemistry & Pharmacology, Faculty of Health Sciences, Ben-Gurion University of the Negev, Beer Sheva 8410501, Israel

**Keywords:** gasoline vapors, vitamin D_3_, turmeric, dietary supplements

## Abstract

The inhalation of gasoline vapors (GV) is associated with developing various pathologies. Particularly, oil refinery and gas station workers are at a greater risk of developing lung cancer, kidney cancer, bladder cancer, and hematological disorders, including acute myeloid leukemia. Therefore, preventing the harmful effects of GV and alleviating their consequences appear to be important and timely issues. In this study, we investigated the potential of vitamin D_3_, turmeric powder, and their combination to ameliorate the toxicity of gasoline fumes in rats. Separate groups of animals fed with a standard rodent diet, with or without the supplementation of vitamin D_3_ (750 IU/kg body weight) and/or turmeric powder (0.5%, *w*/*w*, in food), were untreated or treated with GV (11.5 ± 1.3 cm^3^/h/m^3^/day) for 30, 60, or 90 days. Changes in the body weight were monitored weekly. Histological, biochemical, and hematological parameters were determined at the end of each treatment period. While the exposure of rats to GV resulted in a time-dependent reduction in body weight, supplementation with vitamin D_3_, but not with turmeric root powder or their combination, partially prevented weight loss. Macroscopical and histological analyses showed pronounced time-dependent changes in the organs and tissues of GV-treated rats. These included alveolar wall collapse in the lungs, the destruction of the lobular structure and hepatocytolysis in the liver, the shrinkage and fragmentation of glomeruli in the kidneys, and the disorganization of the lymphoid follicles in the spleen. However, co-treatment with the nutritional supplements tested, especially vitamin D_3_, noticeably alleviated the above conditions. This was accompanied by a significant improvement in the blood chemistry and hematological parameters. Collectively, our results demonstrate that the harmful effects of environmental exposure to GV can be reduced upon supplementation of vitamin D_3_. The fact that the protective activity of vitamin D_3_ alone was higher than that of turmeric root powder or the combined treatment suggests that combinations of these supplements may not always be more beneficial than each agent applied separately.

## 1. Introduction

Gasoline is the most consumed product of crude oil refining, which contains at least 150 hydrocarbons, including alkanes (60–70%), aromatics (25–30%), and alkenes (6–9%). Gasoline vapors (GV) have been linked to various pathologies, such as lung disorders, hematotoxicity, and encephalopathies [1,2,3,4]. The results of many studies have also shown that employees of gas stations and refineries who are chronically exposed to GV are at a high risk of developing lung, kidney, and bladder cancers [5,6,7], as well as hematological malignancies [8]. Therefore, it is essential to develop protective and preventive measures against the toxic effects of GV. Nevertheless, little attention has been paid to this issue.

To date, several studies have examined the protective actions of some natural agents in preventing or mitigating the harmful effects of GV in rat models in vivo. For instance, supplementing a standard rodent diet with fenugreek seed powder (5%, *w*/*w*) was shown to alleviate pathological changes in the liver and lung biochemical and histological parameters and suppress oxidative stress and inflammation in GV-exposed rats [9,10]. In a similar study, consuming green tea extract (1.5%, *v*/*v* drinking water) or dietary powdered rhizomes of *Curcuma longa* L. (turmeric root powder; 3%, *w*/*w* food) reduced DNA fragmentation in the spleen and liver of mice subjected to GV inhalation [11]. More studies have shown the ability of turmeric root powder to alleviate the harmful effects of other environmental agents and drugs. In particular, the inclusion of turmeric root powder (200 mg/kg body weight) in drinking water [12] or by intragastric gavage [13] was found to protect rats from hepatotoxicity induced by cadmium or carbon tetrachloride, respectively. Likewise, dietary supplementation of turmeric root powder at 2% or 4% (*w*/*w*) reduced renal damage caused by gentamycin. This was associated with decreased plasma levels of renal function markers and an improved antioxidant status in kidney homogenates [14]. In another study, adding turmeric powder to food at 1%, 2%, or 5% (*w*/*w*) attenuated oxidative stress in the gastric, liver, kidney, and heart tissues of rats treated with an ulcerogenic dose of indomethacin [15].

Vitamin D is a multifunctional nutrient produced in the skin through exposure to sunlight and can also be obtained from food. It is becoming increasingly clear that active vitamin D metabolites play a critical role in human health [16]. Vitamin D has been extensively studied for its beneficial effects on various pathologies, including cardiovascular diseases [17], diabetes [18], and infectious diseases [19]. Although vitamins A (400 IU/kg), C (200 mg/kg), and E (200 IU/kg or 400 IU/kg) have reportedly exhibited hepatoprotective activity in the rat model of GV toxicity [20,21], the potential ability of vitamin D to prevent harmful effects of GV has not yet been studied. However, a number of animal studies have shown that vitamin D protects against other environmental factors, toxicants, and drugs [22]. For instance, intramuscular injections of vitamin D_3_ (1000 IU/kg, 3 days a week) alleviated the damage to the liver [23], kidneys, and testicles [24] in rats consuming lead in the drinking water. This was accompanied by lowering the levels of oxidative stress and pro-inflammatory markers and increasing the expression of antioxidant and anti-inflammatory markers and vitamin D- and Ca^2+^-related regulatory molecules in the damaged tissues [23,24]. In a similar study, intramuscular injections of vitamin D_3_ (600 IU/kg, 3 times a week) and/or oral supplementation of calcium (100 mg/kg, 5 times a week) protected rats from cadmium hepatotoxicity. Notably, the two agents positively co-operated when applied together [25]. It has also been reported that the intraperitoneal administration of vitamin D_3_ (20 IU/kg, daily) had a protective effect against carbon tetrachloride-induced nephrotoxicity in rats. This was manifested by the restoration of serum levels of renal markers (urea and creatinine) and the recovery of histopathological lesions in the kidneys [26]. El-Boshy et al. [27] have investigated the prophylactic and therapeutic activities of intraperitoneally administered vitamin D_3_ against paracetamol-induced hepatorenal damage. They reported that two rounds of vitamin D_3_ injections at 1000 IU/kg/day (5 days/week) before and another round after paracetamol poisoning showed better protective effects compared to a single round of vitamin D_3_ at a higher dose (3000 IU/kg/day; 5 days) just post-paracetamol intoxication.

Despite the accumulated evidence that supplementation with turmeric root powder or vitamin D_3_ alone can protect against chemical and drug toxicants in several animal models, it remains unclear whether these agents can co-operate against GV toxicity. Therefore, in the present study, we investigated whether supplementation with vitamin D_3_, turmeric powder, or their combination could ameliorate pathological changes in rats exposed to GV. Our results demonstrated that the consumption of vitamin D_3_ significantly improved the macroscopical organ appearance, as well as histological, biochemical, and hematological parameters in GV-exposed rats. The protective effects of turmeric or its combination with vitamin D_3_ were found to be less pronounced. These findings suggest that the harmful effects of environmental exposure to GV can be reduced upon supplementing the diet with vitamin D_3_.

## 2. Results

**Figure 1:**
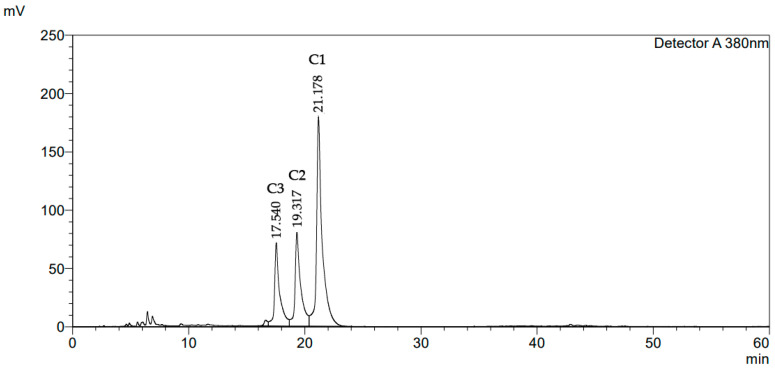
HPLC chromatogram of the methanolic turmeric root powder extract. C1, curcumin; C2, demethoxycurcumin; C3, bisdemethoxycurcumin.

Separate groups of unexposed and GV-exposed rats were supplemented with vitamin D_3_, turmeric root powder, or their combination for 30–90 days, as described in Materials and Methods (Section 4.2). Control (untreated) and GV-only-treated rats were not given any supplements. Vitamin D_3_ was administered orally (750 IU/kg body weight) 6 days a week. Commercial turmeric root powder (Kevala International LLC; certified by the United States Department of Agriculture) was mixed with rodent food at 0.5% (*w*/*w*) and provided ad libitum. The phytochemical analysis of this product (Figure 1 and Table 1) showed that the quantities of the major curcuminoids were similar to those previously reported for different commercial turmeric powders: curcumin (62–90 mg/g), demethoxycurcumin (9–23 mg/g), and bisdemethoxycurcumin (0.3–14 mg/g) [28]. Consistent with previous studies [29,30], our turmeric product also contained other phytochemicals, such as flavonoids, tannins, alkaloids, and saponins (Table 1).

### 2.1. Changes in the Body Weight of Rats Supplemented with Vitamin D_3_, Turmeric Powder, or Their Combination, with and without Exposure to Gasoline Vapors

**Figure 2:**
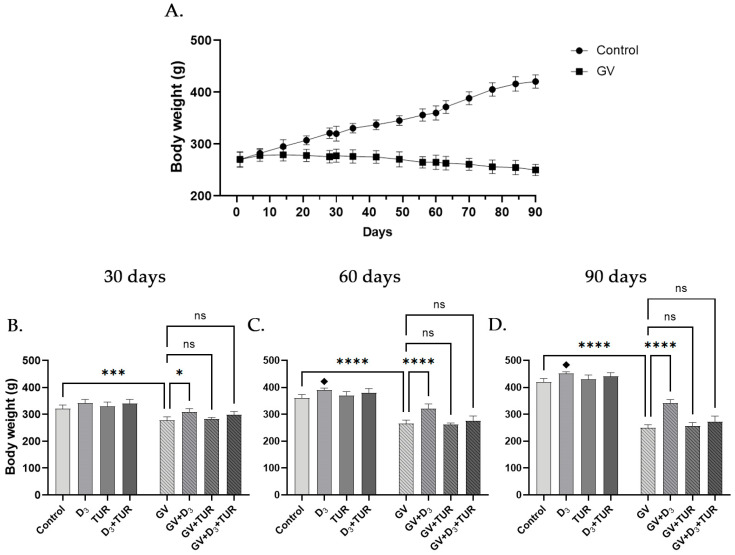
Changes in the body weight of unexposed and GV-exposed rats following supplementation with vitamin D_3_, turmeric powder, or their combination. Rats from all groups (15 rats/group) were weighted weekly starting from Day 0. (**A**) Comparison of the body weight gain between control and GV-alone-treated group. (**B**–**D**) Five rats from the indicated groups were weighed on days 30, 60, and 90. Data are mean ± SD. One-way ANOVA followed by Tukey’s post hoc multiple comparisons test. *, *p* < 0.05; ***, *p* < 0.001; ****, *p* < 0.0001 significant differences between the indicated groups; ♦, *p* < 0.05 vs. untreated control group; ns, not significant.

As demonstrated in Figure 2A–D, the exposure of rats to GV resulted in a time-dependent reduction in body weight. In particular, a 15.2%, 31.0%, and 41.4% reduction was observed following 30, 60, and 90 days of exposure, respectively, compared with the control group (Figure 2B–D). GV-treated rats supplemented with vitamin D_3_ alone, but not with turmeric powder or their combination, exhibited a significantly less pronounced weight loss than the GV alone group. Interestingly, vitamin D_3_ treatment without GV exposure also resulted in a small but significant increase in body weight compared to the control (Figure 2B–D). These data indicate that, among the supplements used, only vitamin D_3_ had a beneficial effect against GV-induced weight loss.

### 2.2. Macroscopical and Histological Features of the Lungs, Liver, Kidneys, and Spleen of Rats Supplemented with Vitamin D_3_, Turmeric Powder, or Their Combination, with and without Exposure to Gasoline Vapors

**Figure 3:**
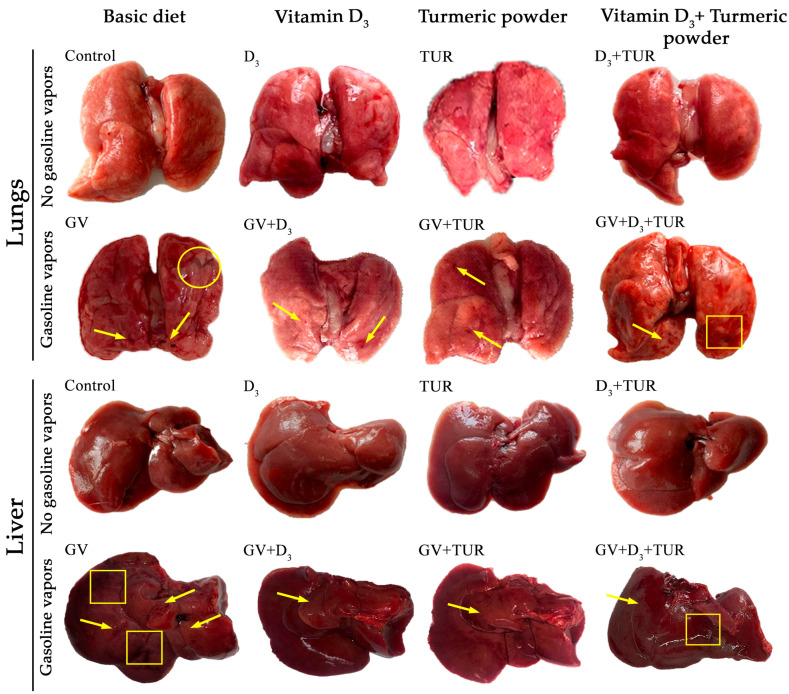
Changes in the macroscopical appearance of the lungs and the liver of unexposed and GV-exposed rats supplemented with vitamin D_3_, turmeric powder, or their combination. Following the indicated treatments, the organs were excised on Day 60 and photographed. Representative images of the organs from one out of five rats in each group are shown. *Arrows*—tissue hemorrhages; *squares*—tissue discoloration; *circles*—pleural exudate.

**Figure 4:**
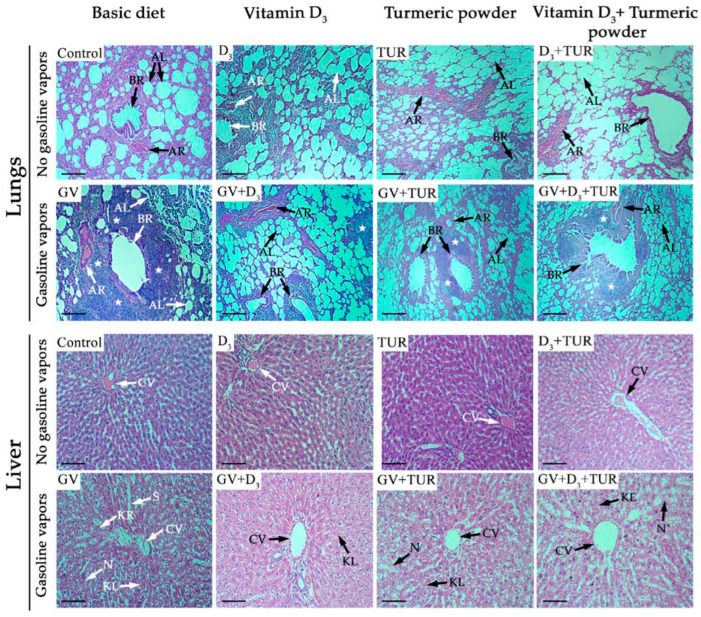
Histological changes in the lungs and the liver of unexposed and GV-exposed rats supplemented with vitamin D_3_, turmeric powder, or their combination. H&E-stained tissue sections were prepared from the rats subjected to the indicated treatments for 60 days. Representative images of the sections from one out of five rats in each group are shown. **AL**—alveoli; **BR**—bronchioles; **AR**—arterioles; **CV**—central vein; **N**—necrosis; **KR**—karyorrhexis; **KL**—karyolysis; **S**—sinusoids; *stars*—cellular infiltrates. Magnification, ×100. Scale bars, 50 µm.

Organs were excised from all groups of rats (5 rats/group) following 30, 60, and 90 days of treatments, photographed, and fixed in neutral buffered formalin for further histological examination. Hematoxylin and eosin (H&E)-stained tissue sections were analyzed under a light microscope. Figure 3 and Figure 4 and Supplementary Figures S1 and S2 present the results of the macroscopical and histological analyses, respectively, obtained at the middle time-point of our experiment (Day 60). These data exemplify the toxic effects of GV and the protective effects of the supplements on different organs and tissues.

#### 2.2.1. Macroscopical Analysis

In rats exposed to GV alone, the lungs were swollen, with uneven surfaces and a brownish tint (Figure 3, lungs). Multiple hemorrhages appeared in both lungs (*arrows*), and most animals had pleural exudates (*circles*). The administration of vitamin D_3_, turmeric powder, or their combination largely protected the lungs from the edema, though small sporadic hemorrhages (*arrows*) could still be observed. In contrast to the rats supplemented with vitamin D_3_ alone, the lungs of the animals receiving turmeric powder or its combination with vitamin D_3_ displayed some surface unevenness and darkened areas (*squares*). The areas of discoloration were more frequent in the lungs of the combination-treated animals compared to the turmeric powder group.

Similar to the changes observed in the lungs, the liver in GV-treated rats was swollen and had a rough dark-brown surface without a natural luster (Figure 3, liver). Multiple hemorrhages could primarily be seen in the left and right lateral lobes and the caudate lobe (*arrows*). Areas of unusually darkened tissue were noticed mainly in the right and medial lobes (*squares*). Rats receiving dietary supplements displayed less dramatic overall changes in the liver compared to the GV-only group. Particularly, organ edema was less pronounced, and only small petechial hemorrhages were noticed, primarily in the caudal lobe (*arrows*). The livers from the combination-treated group still displayed some darkened surface regions (*squares*).

A macroscopical examination of the kidneys showed that treatment with GV resulted in significant kidney swelling, with a less noticeable bean-like shape compared to the untreated control group. In most rats of this group, the left kidney was more enlarged than the right one (Supplementary Figure S1, kidneys). Both kidneys had an indurated texture and a rough, dark-brown surface. Supplementation with vitamin D_3_ alone markedly reduced swelling and tissue density and normalized the surface color of the kidneys. The improvement in the groups receiving turmeric powder or its combination with vitamin D_3_ was somewhat less evident compared to the vitamin-D_3_-alone-treated rats. The exposure to GV led to the enlargement, swelling, loss of the ribbon-like shape, and darkening of the spleens. However, dietary supplements, particularly vitamin D_3_ alone, partially protected from this harmful impact of GV (Supplementary Figure S1, spleen).

In the absence of GV, supplementation with vitamin D_3_, turmeric powder, or their combination did not significantly affect the macroscopical features of the organs evaluated in this study (Figure 3 and Supplementary Figure S1). Of note, in both GV-treated and untreated animals supplemented with turmeric root powder or its combination with vitamin D_3_, all the organs, except the spleen, had a yellowish tint (presumably due to the curcuminoid-enriched diet). This is consistent with the previous reports demonstrating a yellowish skin color in animals and humans following turmeric consumption [31,32].

#### 2.2.2. Histological Analysis

To investigate the influence of GV and dietary supplements on the lungs, liver, kidneys, and spleen in more detail, we performed a histological analysis of hematoxylin and eosin (H&E)-stained tissue sections of the above organs (Figure 4 and Supplementary Figure S2). The following changes were detected in the lung tissue of GV-exposed rats compared to the control group (Figure 4, lungs). A large part of the alveoli appeared to be collapsed (*arrow* AL). The inner wall of some of the bronchioles was found to be detached (*arrow* BR), and arteriole walls were thickened, resulting in the narrowing of the lumen (*arrow* AR). In addition, there was massive cellular, likely leukocytic, infiltration in peribronchial and perivascular areas (*stars*), which was associated with the loss of the alveolar architecture. Supplementation with vitamin D_3_, turmeric powder, or their combination partially improved the structure of the lung tissue to a varying extent compared to the GV-alone-treated group. Specifically, the rats receiving vitamin D_3_ alone displayed healthier alveoli and a better-preserved wall structure of the bronchioles compared to the groups supplemented with turmeric powder or its combination with vitamin D_3_ (Figure 4, lungs). Furthermore, there were smaller areas of leukocytic infiltration in the lungs of vitamin-D_3_-supplemented rats compared to the other groups.

Liver tissue sections from the rats exposed to GV (Figure 4, liver) demonstrated profound structural changes, such as disorganized regular radiating rows of hepatocytes, the appearance of necrotic areas (*arrow* N), karyolysis (*arrow* KL), and karyorrhexis (*arrow* KR). In addition, we observed pronounced damage to the central vein walls (*arrow* CV) and sinusoidal dilatation (*arrow* S). However, the administration of vitamin D_3_ largely alleviated the above pathological changes. The addition of turmeric powder and its combination with vitamin D_3_ was less effective in ameliorating GV toxicity than the vitamin D_3_ alone group (Figure 4, liver).

Unlike the kidney tissue sections of control rats (Supplementary Figure S2, kidneys), the ones from GV-exposed rats showed the shrinkage and fragmentation of glomeruli (*arrow* G) with a dramatic reduction in the Bowman’s capsule space (*arrow* BC). Furthermore, the renal tubules were much narrower than those of the control group (*arrow* RT), and interstitial hemorrhages could be seen (*arrow* H). Remarkably, supplementation with vitamin D_3_ led to a marked protection of the tissue structure that appeared almost normal, except for occasional small hemorrhages. A certain amelioration of GV-induced changes was also observed in rats receiving turmeric powder or its combination with vitamin D_3,_ but the tissue structure was less preserved compared to the vitamin-D_3_-alone group (Supplementary Figure S2, kidneys).

The analysis of the spleen sections (Supplementary Figure S2, spleen) showed that treatment with GV resulted in an extensive disruption of the lymphoid follicle (LF) structure with a poorly distinguishable mantle region (*arrow* M) and marginal zone (MZ). The lymphoid follicles were barely distinct from the red pulp (RP) and periarterial lymphocytic sheath (P). The administration of vitamin D_3_ or turmeric powder led to a better-preserved structure of the lymphoid follicles with more distinct mantle regions, while, in the combination-treated rats, the protection of the spleen tissue was less noticeable (Supplementary Figure S2, spleen).

In summary, the exposure to GV resulted in time- and organ-dependent pathological changes at the histological level that were ameliorated to varying degrees upon supplementation with vitamin D_3_, turmeric powder, or their combination. No significant differences in tissue structure were observed between the control rats and those administered with the tested supplements in the absence of GV.

### 2.3. Blood Chemistry Analysis of Rats Supplemented with Vitamin D_3_, Turmeric Powder, or Their Combination, with and without Exposure to Gasoline Vapors

To further explore the influence of GV and dietary supplements on the liver and kidneys, we determined the functional status of these organs by measuring the serum concentrations of the corresponding biomarkers. Increased serum levels of alanine aminotransferase (ALT) and aspartate aminotransferase (AST) indicate liver damage, while increased creatinine and urea concentrations point to the deterioration of the kidney function. Decreases in serum total protein and glucose levels may result from malnutrition and impaired liver and kidney functions [33].

**Figure 5:**
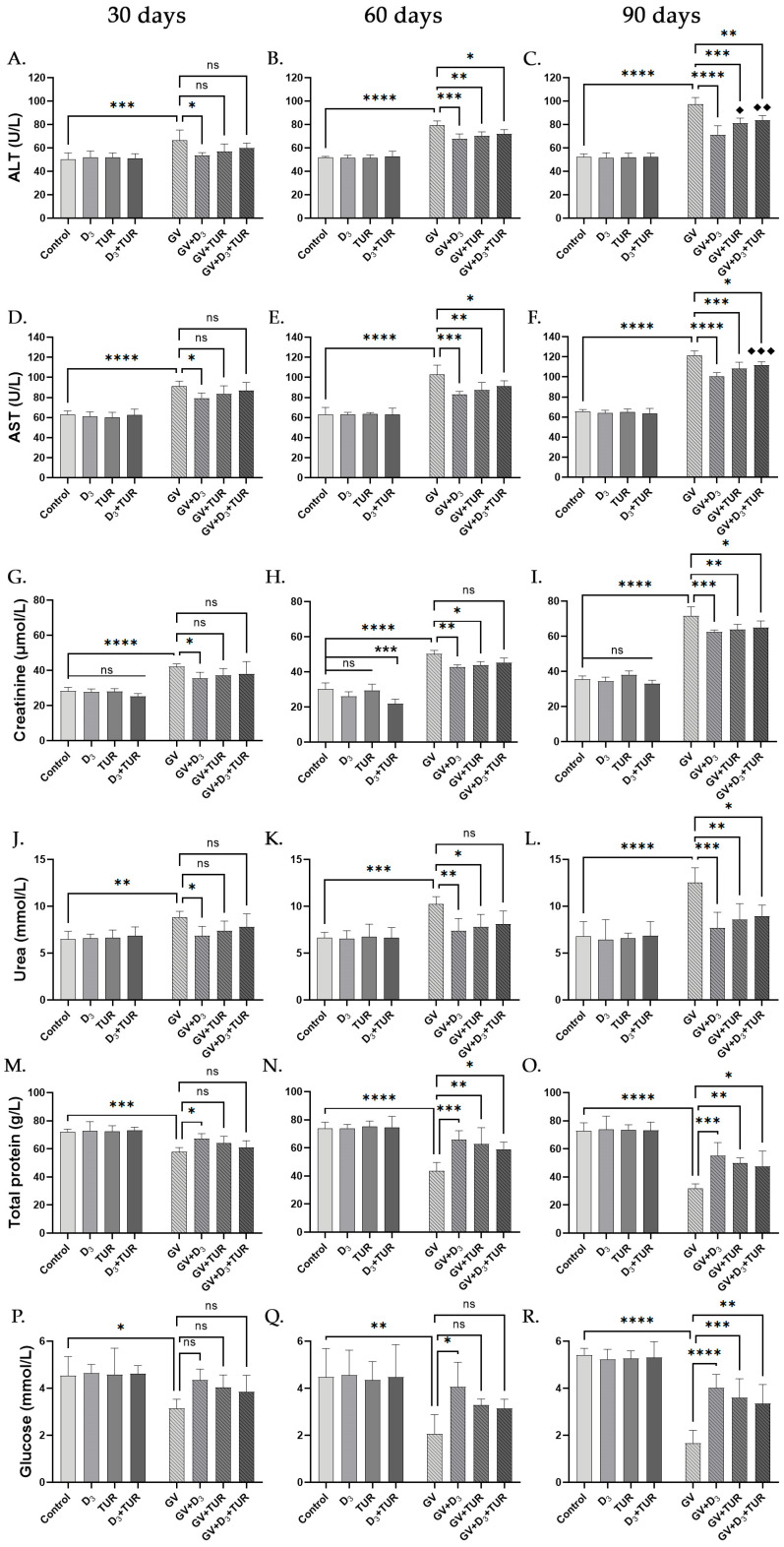
Biochemical changes in the blood of unexposed and GV-exposed rats supplemented with vitamin D_3_, turmeric powder, or their combination. Peripheral blood samples from five rats of the indicated groups collected on days 30 (**A**,**D**,**G**,**J**,**M**,**P**), 60 (**B**,**E**,**H**,**K**,**N**,**Q**), and 90 (**C**,**F**,**I**,**L**,**O**,**R**) were analyzed. Data are mean ± SD. One-way ANOVA followed by Tukey’s post hoc multiple comparisons test. *, *p* < 0.05; **, *p* < 0.01; ***, *p* < 0.001; ****, *p* < 0.0001 significant differences between the indicated groups; ♦, *p* < 0.05; ♦♦, *p* < 0.01; ♦♦♦, *p* < 0.001 vs. GV + D_3_ group; ns, not significant.

The exposure of rats to GV for 30, 60, and 90 days resulted in a marked and time-dependent elevation of the serum levels of the liver markers, ALT (by 32.6%, 53.1%, and 86.7%, respectively; Figure 5A–C) and AST (by 45.1%, 63.1%, and 85.2%, respectively; Figure 5D–F), as compared with the corresponding control group. However, the administration of vitamin D_3_ alone led to a significant decline in serum levels of both ALT (by 19.3%, 14.6%, and 26.9%, respectively; Figure 5A–C) and AST (by 13.4%, 19.6%, and 17.2%, respectively; Figure 5D–F), compared to the corresponding GV group. Supplementation with turmeric powder or its combination with vitamin D_3_ tended to be less effective than vitamin D_3_ alone (e.g., Figure 5C,F).

Time-dependent increases in creatinine and urea serum levels were also a characteristic of GV-exposed rats. Specifically, following 30, 60, and 90 days of treatment, creatinine and urea levels were elevated by 49.3%, 66.3%, and 102.9%, and by 35.6%, 54.3%, and 84.2%, respectively, relative to the corresponding control group (Figure 5G–L). The co-administration of the supplements and their combination significantly improved the levels of both protein metabolites, the combined treatment tending to be the least effective. Treatment with vitamin D_3_ resulted in a minor (by 13–16%) but significant reduction in creatinine levels at all the time points (Figure 5G–I), while the decreases in urea levels in this group were time-dependent and more pronounced (by 22.3%, 27.8%, and 38.7% vs. GV group, respectively; Figure 5J–L).

Exposure to GV for 30, 60, and 90 days led to a substantial reduction in the serum concentrations of the total protein (by 19.2%, 40.8%, and 56.5%, respectively) and glucose (by 31.0%, 54.1%, and 69.2%, respectively) relative to the corresponding controls. However, supplementation with vitamin D_3_ alone significantly reversed these harmful effects by elevating both the total protein levels (by 16.0%, 50.7%, and 74.1%, respectively; Figure 5M–O) and glucose levels (by 39.0%, 98.0%, and 140.2%; respectively; Figure 5P–R) compared to the corresponding GV group. The diets fortified with turmeric powder or its combination with vitamin D_3_ had similar, though less efficient, protection (Figure 5M–R). Of note, vitamin D_3_ supplementation was capable of essentially restoring serum urea and glucose concentrations in GV-treated rats approximately up to the untreated control levels throughout the entire experimental period (Figure 5J–L,P–R).

The supplements given to GV-untreated rats did not cause adverse effects on the blood chemistry parameters (Figure 5) and even tended to reduce serum creatinine levels in these animals with time, mainly on Day 60 (Figure 5G–I).

### 2.4. Hematological Analysis of Rats Supplemented with Vitamin D_3_, Turmeric Powder, or Their Combination, with and without Exposure to Gasoline Vapors

**Figure 6:**
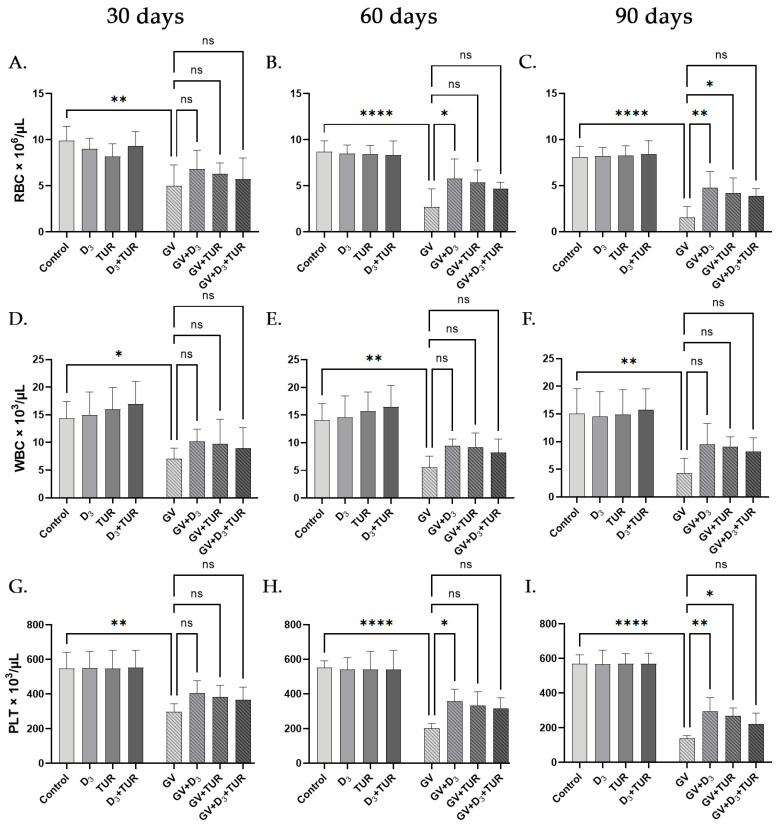
Changes in complete blood counts of unexposed and GV-exposed rats supplemented with vitamin D_3_, turmeric powder, or their combination. Peripheral blood samples from five rats of the indicated groups collected on days 30 (**A**,**D**,**G**), 60 (**B**,**E**,**H**), and 90 (**C**,**F**,**I**) were analyzed. Data are mean ± SD. One-way ANOVA followed by Tukey’s post hoc multiple comparisons test. *, *p* < 0.05; **, *p* < 0.01; ****, *p* < 0.0001 significant differences between the indicated groups; ns, not significant.

A complete blood count (CBC) was performed in the peripheral blood samples to examine the influence of GV inhalation and dietary supplements on the hematological parameters of experimental rats. GV exposure for 30, 60, and 90 days resulted in a highly significant and time-dependent decrease in the red blood cells (RBC) by 49.8%, 68.6%, and 81.2%, respectively, compared to the control group (Figure 6A–C). Similar data were obtained by measuring hematocrit (HCT) and hemoglobin (HGB) levels (Supplementary Figure S3A–C and S3D–F, respectively). Importantly, all the above parameters were partially recovered as a result of vitamin D_3_ supplementation in a time-dependent manner, as follows: The levels of RBC were elevated by 36.9%, 111.7%, and 214.5% (Figure 6A–C); HCT by 29.0%, 136.8%, and 207.2% (Supplementary Figure S3A–C), and HGB by 39.7%, 147.6%, and 243.2% (Supplementary Figure S3D–F) after 30, 60, and 90 days, respectively, compared to the corresponding GV group.

GV inhalation also caused significant and time-dependent decreases in white blood cell (WBC) counts, including granulocyte (GRA) and lymphocyte (LYM) percentages and platelet counts (Figure 6D–F,G–I; Supplementary Figure S3G–L). For instance, after 30, 60, and 90 days, WBC counts were reduced by 50.7%, 60.5%, and 71.1%, respectively (Figure 6D–F), and platelet counts by 45.6%, 63.5%, and 75.7%, respectively (Figure 6G–I), compared to the control group. Similar to the protective action of vitamin D_3_ on the red blood cell parameters, we observed a significant increase in WBC counts (by 43.8%, 71.1%, and 119%; Figure 6D–F) and platelet counts (by 36.0%, 77.2%, and 114.5%; Figure 6G–I), respectively, in vitamin-D_3_-treated groups compared to the corresponding GV groups. A comparable reversal of GV-induced granulocytopenia (Supplementary Figure S3G–I) and lymphopenia (Supplementary Figure S3J–L) by vitamin D_3_ supplementation was also detected. The dietary administration of turmeric powder or its combination with vitamin D_3_ had similar but less noticeable effects on the above hematological parameters tested in GV-exposed rats. Neither supplementation type significantly influenced GV-untreated rats (Figure 6 and Supplementary Figure S3).

## 3. Discussion

In this study, we investigated the potential of oral supplementation with vitamin D_3_, turmeric powder, and their combination to reduce chronic GV toxicity in rats. A number of animal studies have shown that vitamin D protects against the harmful influence of different environmental factors, toxic compounds, and drugs, e.g., lead [23,24], cadmium [25], carbon tetrachloride [26], and paracetamol [27]. However, to the best of our knowledge, the effects of vitamin D against GV toxicity have not yet been reported, and only one publication so far has described the use of dietary turmeric powder in an animal model of GV poisoning [11].

In the present study, we demonstrated, for the first time, that oral treatment with vitamin D_3_ at a moderate dose of 750 IU/kg (6 days/week) for 30–90 days alleviated GV-induced toxicity in rats. This was associated with an improvement in the general condition of the animals manifested by a better appearance and less pronounced reduction in the body weight, which appeared to stabilize over the time course of supplementation relative to the continuing weight loss in GV-treated animals (Figure 2). Vitamin D_3_ treatment resulted in at least a partial preservation of the intact organ appearance (Figure 3 and Supplementary Figure S1) and tissue structure (Figure 4 and Supplementary Figure S2) of the lungs, liver, kidneys, and spleen. Furthermore, there was a significant improvement in serum levels of liver and kidney functional biomarkers and glucose (Figure 5), as well as a partial restoration of hematological parameters, such as red blood cell, white blood cell, and platelet counts (Figure 6 and Supplementary Figure S3). A similar but less effective protection was observed following dietary supplementation with turmeric powder at a relatively lower dose (0.5%, *w*/*w*; 6 days/week) compared to those used in previous studies (e.g., [11,14,15]). These protective effects of vitamin D_3_ and turmeric are consistent with their known antioxidant, anti-inflammatory, and immunomodulatory activities [34,35,36,37,38].

Although the combined effects of vitamin D_3_ and turmeric/curcumin on GV toxicity have not been previously determined, several studies have reported a positive co-operative activity of these agents in animal models of other pathologies. In particular, the oral administration of a nanoencapsulated combination of vitamin D_3_ (16 IU/day) and curcumin (4 mg/kg) was found to be an effective anti-inflammatory adjuvant treatment of rheumatoid arthritis in rats [39]. In a similar model in mice, a diet enriched with vitamin D_3_ (10,000 IU/kg food) and omega-3-fatty acids (10 g/kg food) combined with the oral supplementation of a highly bioavailable form of curcumin (100 mg/kg) markedly reduced the severity of collagen-induced arthritis, and delayed the onset and slowed the progression of the disease [40]. In another study, the oral administration of the formulation containing 33.26% total curcuminoids, 3.47% lutein, 0.7% zeaxanthin, and 930 IU vitamin D_3_ (200 mg/kg) alleviated the symptoms of the dry eye condition in rats [41]. Attia et al. [42] have reported that the combination of curcumin, the active hormonal form of vitamin D_3_ (1α,25-dihydroxyvitamin D_3_; 1,25(OH)_2_D_3_), and the anticancer drug paclitaxel produced a synergistic cytotoxic effect on human MCF-7 breast cancer cells in vitro. This study also showed that oral treatment with 50 mg/kg of curcumin and 5000 IU/kg of vitamin D_3_ (3 times/week) resulted in a co-operative reduction in murine Ehrlich ascites carcinoma tumor size in vivo. Similar synergistic activities of 1,25(OH)_2_D_3_ and curcumin or resveratrol were obtained in preclinical in vitro and in vivo models of triple-negative breast cancer [43]. Additionally, we have previously reported that the combination of 1,25(OH)_2_D_3_ and curcumin synergistically induced cell differentiation and a partial G0/G1 cell-cycle arrest in acute myeloid leukemia cells in vitro [44,45].

In contrast to the beneficial effects of the vitamin D_3_ and turmeric/curcumin combinations described above, we unexpectedly observed either no co-operation between these agents or even less adequate protection by their combination relative to the effects of single treatments. In some cases, the co-administration of turmeric powder appeared to diminish or even abolish the protective effect of vitamin D_3_ (e.g., Figure 2, Figure 5 and Figure 6 and Supplementary Figure S3). At this stage, the reason for this apparent antagonism is unclear, even though we used relatively low doses of the two components, which, individually or in combination, had no toxic influence on healthy control rats. It is possible that other turmeric components (flavonoids, tannins, alkaloids, and/or saponins) rather than, or along with, curcuminoids were responsible for suppressing the beneficial effects of vitamin D_3_. One mechanistic explanation might be that curcuminoids and other polyphenols (flavonoids and tannins; see Table 1), known as antioxidant and antiinflammatory agents, can also exhibit dose-dependent pro-oxidant activities under specific conditions [46,47,48,49,50,51].

Interestingly, data from several large-scale human intervention studies also demonstrated the adverse effects of the known antioxidant β-carotene, alone or in combination with vitamin A or E, on cancer incidence and all-cause mortality. For instance, the Alpha-Tocopherol, Beta-Carotene Cancer Prevention (ATBC), and the Beta-Carotene and Retinol Efficacy Trial (CARET) studies demonstrated that cigarette smokers given supplements of β-carotene and either vitamin E [52] or vitamin A [53,54] had an increased lung cancer incidence, as well as the overall and cardiovascular mortality rates. Further, a meta-analysis of data from 68 randomized trials revealed that supplementation with β-carotene, vitamin A, or vitamin E, alone or in combination, was associated with a significantly increased risk of colon cancer and overall mortality [55]. The cause of the increased mortality in the studies reported above remains unclear, though it was suggested that, at the applied doses, some antioxidant compounds, such as β-carotene and vitamin C, might act as pro-oxidants [53,54]. The fact that, in both ATBC and CARET studies, similar negative consequences occurred in the β-carotene-containing arms suggests that β-carotene was the agent responsible for the adverse effects [54].

More studies are needed to characterize further the protective action of vitamin D_3_ against GV toxicity and to determine the mechanism of this effect. Although our data showed a certain antagonistic relationship between vitamin D_3_ and turmeric powder supplements, examining other natural agents that might positively co-operate with this multifaceted vitamin against GV toxicity would be useful.

## 4. Materials and Methods

### 4.1. Experimental Rats

This study was carried out in the animal facility of the al-Farabi Kazakh National University in accordance with the protocol approved by the ethical commission of the RSE “Institute of Human and Animal Physiology” CS MES, Republic of Kazakhstan (No. 12-28 of 3 February 2023). In the experiment, 120 inbred male albino rats (3 months old) with an initial weight of 270.8 ± 12.6 g were used. The animals were housed at 15 rats/cage and had free access to drinking water and a standard rodent diet (SS R 50258-92, Krupy Vostoka, Oskemen, Kazakhstan) containing 200 IU/kg vitamin D. Before the experiment, the animals were quarantined for 14 days.

### 4.2. Experimental Protocols and Sample Collection

Rats were randomly divided into 8 groups (15 rats/group) and treated as follows:

Gr.1—Control: untreated.
Gr.2—D_3_: oral vitamin D_3_ in a liquid form (Detrimax^®^ Baby, Curtis Health Caps, Przeźmierowo, Poland) at a daily dose of 750 IU/kg.
Gr.3—TUR: turmeric root powder (Kevala International LLC, Dallas, TX, USA) mixed with standard diet at 0.5% (*w*/*w*).
Gr.4—D_3_ + TUR: oral vitamin D_3_ and dietary turmeric powder.
Gr.5—GV: exposure to GV (11.5 ± 1.3 cm^3^/h/m^3^/day).
Gr.6—GV + D_3_: exposure to GV and oral vitamin D_3_.
Gr.7—GV + TUR: exposure to GV and dietary turmeric powder.
Gr.8—GV + D_3_ + TUR: exposure to GV, oral vitamin D_3_, and dietary turmeric powder.

The rats were followed for 90 days. Changes in the body weight were recorded weekly. Five rats from each group were subjected to laboratory tests after 30, 60, and 90 days as follows. Animals were anesthetized by inhalation of a lethal dose of ether. Blood (~1 mL) was immediately sampled from the superior vena cava into vacuum tubes for blood chemistry tests (without anticoagulant) and hematological analysis (with ethylenediaminetetraacetic acid as an anticoagulant). Following termination, rats were autopsied, and internal organs were visually examined. Liver, lungs, kidneys, and spleen were then excised, macroscopically evaluated, photographed by a Canon Zoemini S2 digital camera (Canon Inc., Kowloon, Hong Kong, China), and fixed in 10% neutral buffered formalin for the following histopathological analysis.

### 4.3. Phytochemical Analysis of Turmeric Root Powder

#### 4.3.1. Extraction and HPLC Analysis of Curcuminoids

The procedure described by Li et al. [28] was adapted with a minor modification, using purified curcumin, demethoxycurcumin, and bisdemethoxycurcumin (Aktin Chemicals, Inc., Chengdu, China) as standards. Turmeric root powder (0.3 g) was extracted in 30 mL absolute methanol in an ultrasonic bath (KQ5200B, Kunshan Ultrasonic Instrument Co., Ltd., Kunshan, China) at room temperature for 60 min. The extract was filtered through a 0.22 µm filter, and a 15 µL aliquot was subjected to HPLC analysis. A Shimadzu LC-40 HPLC system composed of an LC-40D pump and an SPD-40 UV-VIS detector (Shimadzu Corporation, Kyoto, Japan) was employed. The separation was performed on a ZORBAX Eclipse XDB-C18 column (length, 250 mm; inner diameter, 4.6 mm; 5 µm particle size; Agilent Technologies, Santa Clara, CA, USA) at a constant flow rate of 1 mL/min. The mobile phase comprised acetonitrile and 0.1% formic acid aqueous solution. The following acetonitrile gradient elution program was used: 40–50% for 0–30 min, 50–65% for 30–35 min, 65–70% for 35–42 min, 70% for 42–55 min, and 70–100% for 55–60 min. The eluent absorbance was monitored at 380 nm for quantitative analysis. LabSolutions LC version 5.101 software (Shimadzu, Kyoto, Japan) was used to control the HPLC system and analyze the data. The chromatographic profile of curcuminoids is exemplified in Figure 1.

#### 4.3.2. Quantitative Determination of Phytochemical Constituents of Turmeric Powder

Total flavonoid content was determined by the aluminum chloride colorimetric method, using quercetin as a standard [56]. Total tannin content was analyzed by the thermometric titration method, using gallic acid as a standard [57]. The total content of alkaloids was assessed by the acid dye colorimetry method, using thermopsine as a standard [58]. The total saponin content was measured colorimetrically following cold acetone precipitation, using glycyrrhizic acid as a standard [59].

### 4.4. Exposure to Gasoline Vapor

Rats (Groups 5–8) were subjected to GV inhalation using the protocol described by Uboh et al. [20]. Briefly, 4 cages (15 rats/cage) were placed in separate exposure chambers (110 cm × 90 cm × 110 cm; 1.089 m^3^). Each chamber included two 1000 cm^3^ glass beakers containing 500 cm^3^ liquid gasoline that could freely evaporate at ambient temperature. Animals were exposed to GV for 6 h/day (11.5 ± 1.3 cm^3^/h/m^3^/day), 6 days a week [20], for 30–90 days.

### 4.5. Histopathological Analysis

Formalin-fixed tissue samples were embedded in paraffin blocks. Five to six μm sections were cut by a Technom MZP-01 microtome (Technom, Yekaterinburg, Russia). The tissue slices were dehydrated in a series of decreasing alcohol concentrations and stained with hematoxylin–eosin (H&E; BioVitrum, Saint-Petersburg, Russia). Three random non-overlapping fields of each section were analyzed at 100× magnification on a MicroOptix MX 300 T light microscope equipped with a Vision CAM^®^ V500 digital camera (MicroOptix, Wiener Neudorf, Austria).

### 4.6. Blood Chemistry Analysis

Blood samples were left for 3 h at room temperature for coagulation, followed by centrifugation at 3000× *g* for 5 min. Serum was then analyzed for glucose, creatinine, urea, total protein, alanine transaminase (ALT), and aspartate aminotransferase (AST) using a HumaStar 100 analyzer (Human Diagnostics Worldwide, Wiesbaden, Germany).

### 4.7. Hematological Analysis

Blood samples were analyzed in an Advia-2120i hematology analyzer (Siemens, Munich, Germany) for erythrocyte, white blood cell, lymphocyte, neutrophil and platelet counts, and hemoglobin and hematocrit levels.

### 4.8. Statistical Analysis

The significance of differences between the means was determined using one-way ANOVA followed by a built-in Tukey’s post hoc multiple comparisons test, which compared the mean of each group with the mean of every other group, providing adjusted *p* values. Differences were considered statistically significant at *p* < 0.05. Statistical analysis was carried out using GraphPad Prism 6.0 software (GraphPad, San Diego, CA, USA).

## 5. Conclusions

The results of this study show, for the first time, that the supplementation of vitamin D_3_ for up to 90 days significantly ameliorated GV toxicity in rats. Turmeric root powder alone caused a similar but less effective protection. However, when both supplements were applied together, turmeric root powder appeared to antagonize some of the protective effects of vitamin D_3_. These data suggest that the combined use of these two agents may not always provide an enhanced beneficial activity, at least against the harmful consequences of GV exposure.

## Figures and Tables

**Table 1:** Phytochemical composition of turmeric root powder.

Quantity ^1^	Curcumin	DMC ^2^	BDMC ^3^	Total Flavonoids	Total Tannins	Total Alkaloids	Total Saponins
mg/g dry weight	69.8	31.6	20.9	2.8	27.0	15.8	41.8

^1^ Quantitative phytochemical assays were performed as described in Section 4. ^2^ DMC, demethoxycurcumin; ^3^ BDMC, bisdemethoxycurcumin.

## Data Availability

All experimental data, except those of the macroscopical and histological analyses obtained on Day 30 and Day 90, are presented in this manuscript and Supplementary Materials. The unenclosed data are available upon a reasonable request.

## Supplementary Materials

The following supporting information can be downloaded at: https://www.mdpi.com/article/10.3390/ph17050619/s1, Figure S1: Changes in the macroscopical appearance of the kidneys and the spleen of control and GV-treated rats supplemented with vitamin D_3_, turmeric powder, or their combination; Figure S2: Histological analysis of the kidney and the spleen tissue sections of control and GV-treated rats supplemented with vitamin D_3_, turmeric powder, or their combination; Figure S3: Changes of the complete blood count in the blood of rats supplemented with vitamin D_3_, turmeric powder, or their combination, with and without exposure to gasoline vapors.
