# Supplementary material for: Protective Effects of Dietary Vitamin D3, Turmeric Powder, and Their Combination against Gasoline Intoxication in Rats
Source: Pharmaceuticals (Basel). 2024 May 10;17(5):619. doi: 10.3390/ph17050619 (PMC11124333; doi:10.3390/ph17050619)
Supplement: Supplementary file 1 [file pharmaceuticals-17-00619-s001.zip › pharmaceuticals-2762242-supplementary.pdf]

Protective Effects of Dietary Vitamin D3, Turmeric Powder and Their Combination against Gasoline Intoxication in Rats

Gulfira Yestemirova [1], Zura Yessimsiitova [1#] and Michael Danilenko [2#,*]

[1] Department of Biodiversity & Bioresources, Faculty of Biology and Biotechnology, al-Farabi Kazakh National University, Almaty 050040, Kazakhstan; yestemirova.gulfira@kaznu.kz; zura@kaznu.kz

[2] Department of Clinical Biochemistry & Pharmacology, Faculty of Health Sciences, Ben-Gurion University of the Negev, Beer Sheva 8410501, Israel; misha@bgu.ac.il

[#] Equal contribution

[*] Correspondence: misha@bgu.ac.il; Tel.: +972-8-647-9979.

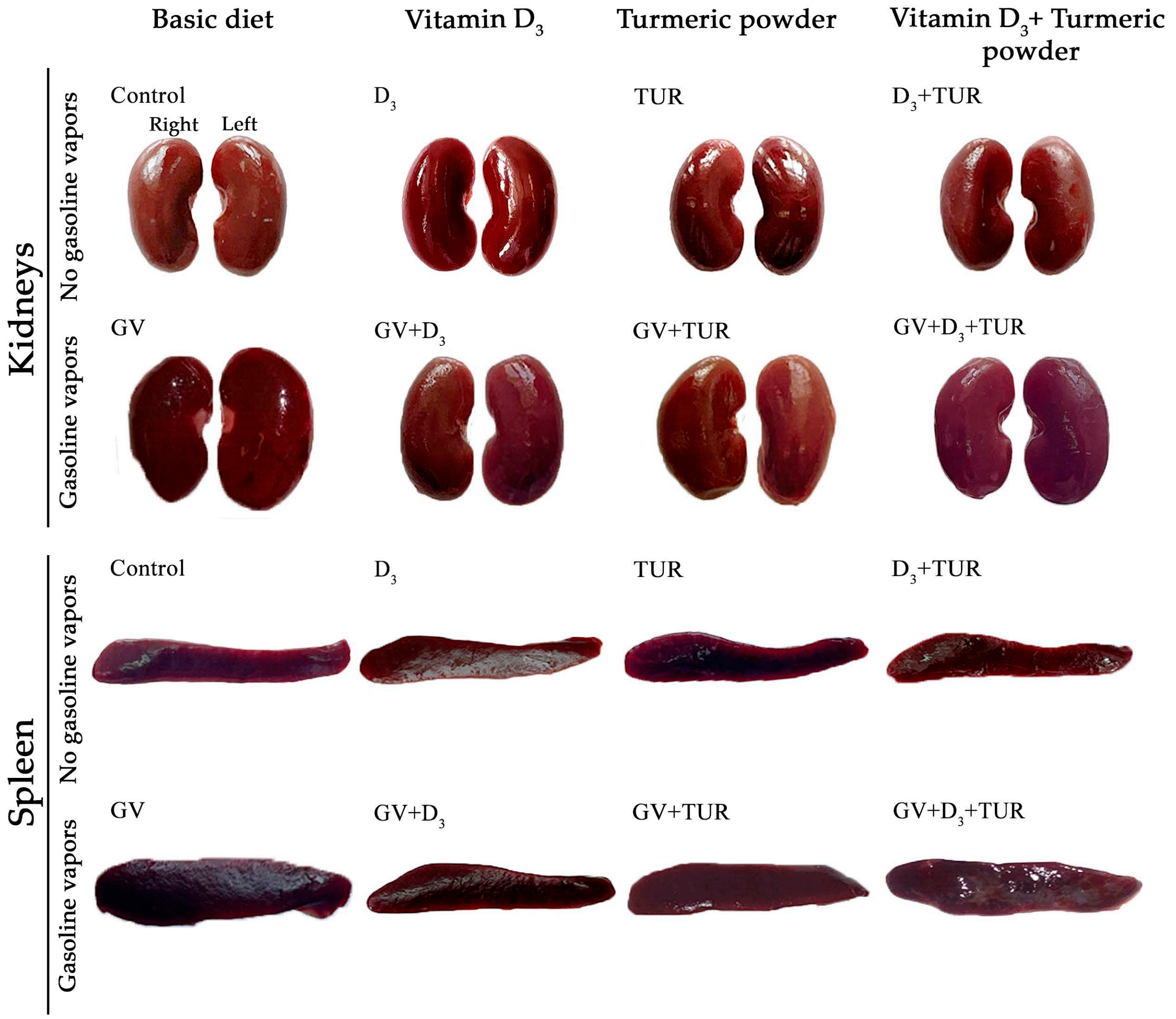

Supplementary Figure S1. Changes in the macroscopical appearance of the kidneys and the spleen of control and GV-treated rats supplemented with vitamin D_3, turmeric powder or their combination. Following the indicated treatments, the organs were excised on day 60 and photographed. Representative images of the organs from one out of five rats in each group are shown.

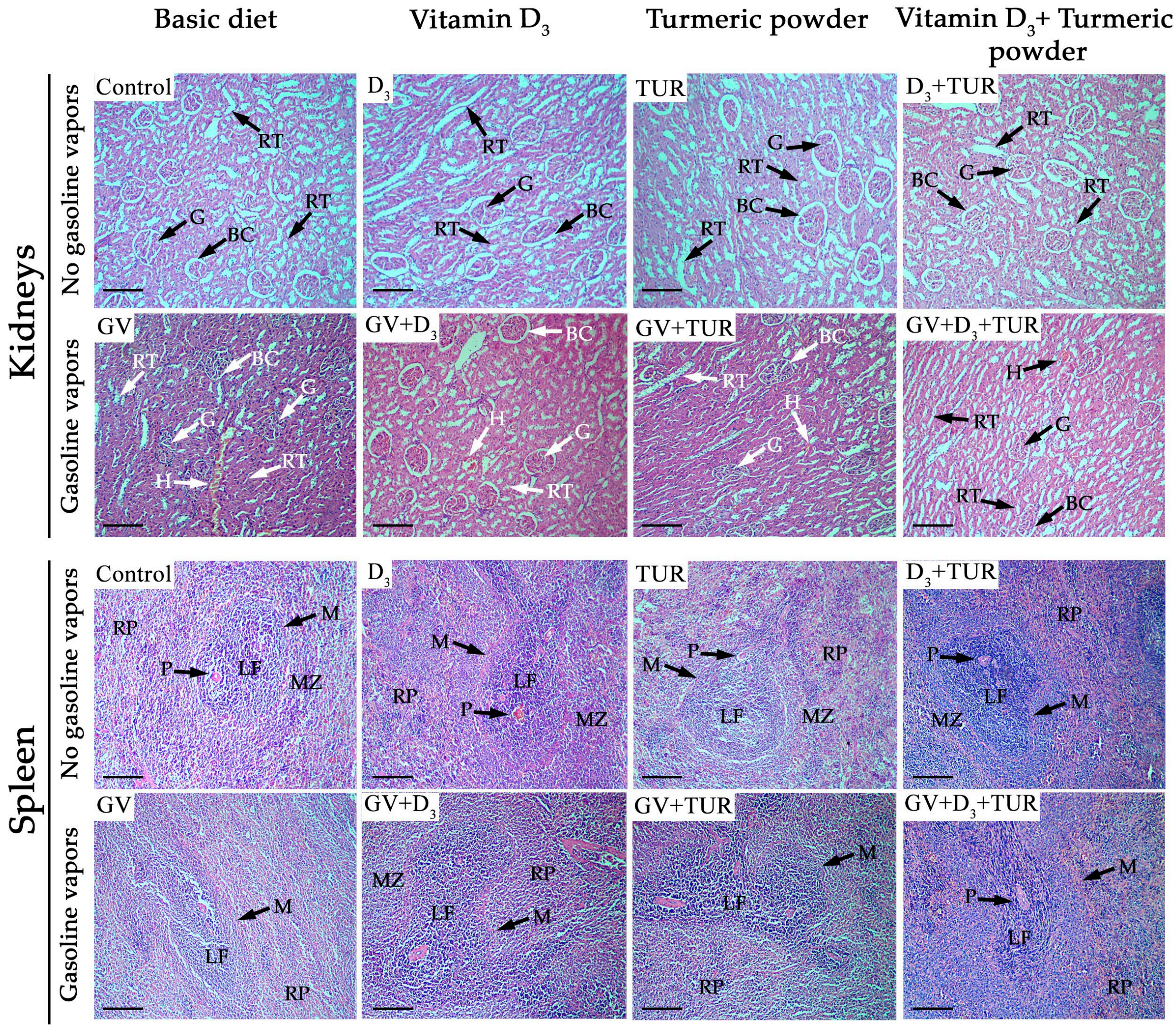

Supplementary Figure S2. Histological changes in the kidney and the spleen of control and GV-treated rats supplemented with vitamin D_3, turmeric powder or their combination. H&E stained tissue sections were prepared from the rats subjected to the indicated treatments for 60 days. Representative images of the sections from one out of five rats in each group are shown. **G** - glomeruli; **BC** - Bowman's capsule; **RT** - renal tubules; **H** – hemorrhages; **LF** - lymphoid follicles; **M** – mantle region; **MZ** – marginal zone; **RP** - red pulp; **P** - periarterial lymphocytic sheath. Magnification ×100. Scale bars, 50 μm.

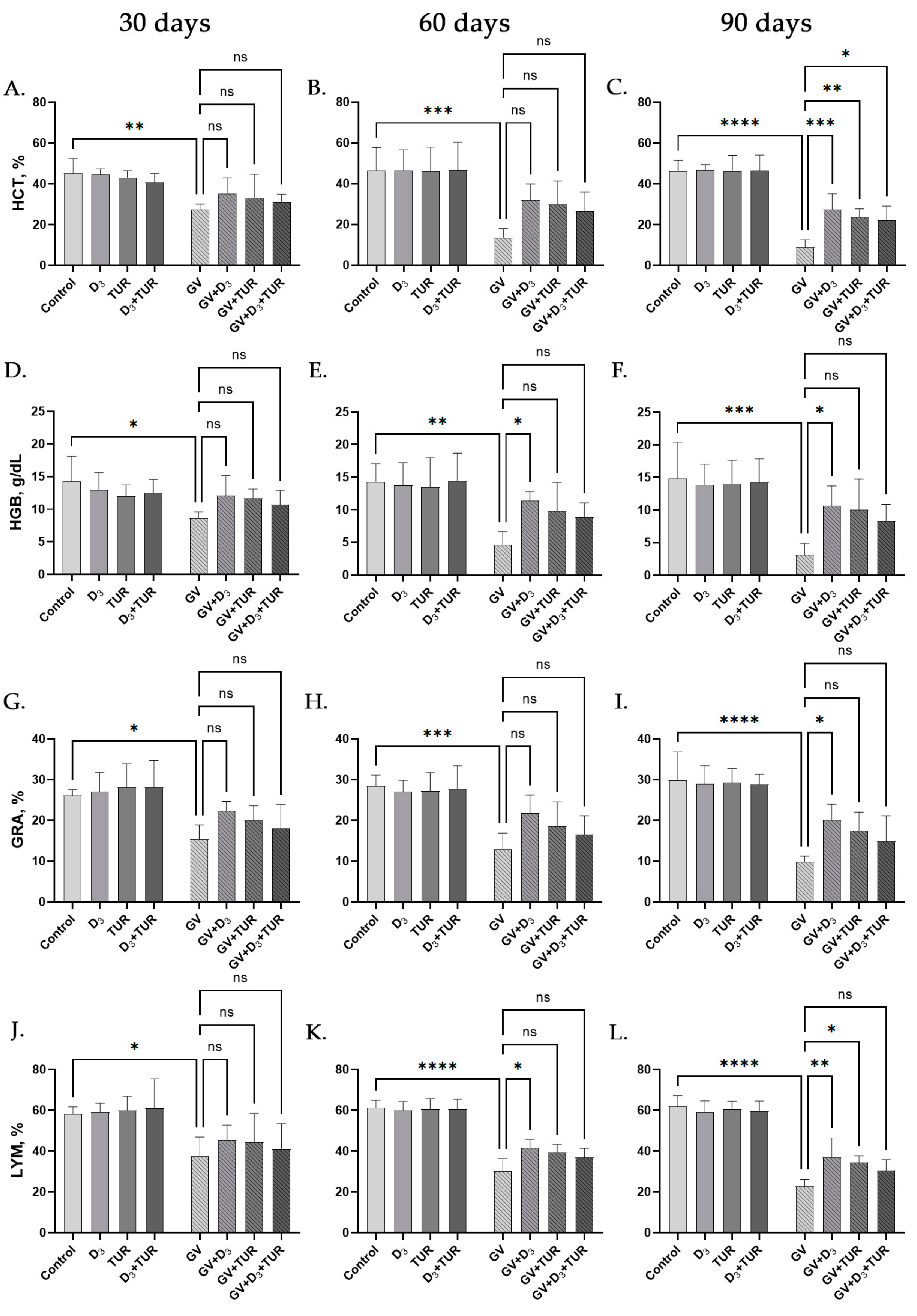

Supplementary Figure S3. Changes in complete blood counts of control and GV-treated rats supplemented with vitamin D_3, turmeric powder or their combination. Peripheral blood samples from five rats of the indicated groups collected on days 30 (A, D, G, J), 60 (B, E, H, K) and 90 (C, F, I, L) were analyzed. Data are mean±SD. One-way ANOVA followed by Tukey's post hoc multiple comparisons test. *, $p < 0.05$; **, $p < 0.01$; ***, $p < 0.001$; ****, $p < 0.0001$ significant differences between the indicated groups; ns – not significant.
